# Mesenchymal stem cell-based therapy for autoimmune-related fibrotic skin diseases—systemic sclerosis and sclerodermatous graft-versus-host disease

**DOI:** 10.1186/s13287-023-03543-w

**Published:** 2023-12-18

**Authors:** Han Yang, Sousan Cheong, Yunfan He, Feng Lu

**Affiliations:** The Department of Plastic and Cosmetic Surgery, Nanfang Hospital, Southern Medical University, 1838 Guangzhou North Road, Guangzhou, 510515 Guangdong China

**Keywords:** Mesenchymal stem cells, Autoimmune-related fibrotic skin diseases, Systemic sclerosis, Sclerodermatous graft-versus-host disease, Chronic graft-versus-host disease, MSC-based therapy

## Abstract

**Background:**

Systemic sclerosis (SSc) and sclerodermatous graft-versus-host disease (Scl-GVHD)—characterized by similar developmental fibrosis, vascular abnormalities, and innate and adaptive immune response, resulting in severe skin fibrosis at the late stage—are chronic autoimmune diseases of connective tissue. The significant immune system dysfunction, distinguishing autoimmune-related fibrosis from mere skin fibrosis, should be a particular focus of treating autoimmune-related fibrosis. Recent research shows that innovative mesenchymal stem cell (MSC)-based therapy, with the capacities of immune regulation, inflammation suppression, oxidation inhibition, and fibrosis restraint, shows great promise in overcoming the disease.

**Main body:**

This review of recent studies aims to summarize the therapeutic effect and theoretical mechanisms of MSC-based therapy in treating autoimmune-related fibrotic skin diseases, SSc and Scl-GVHD, providing novel insights and references for further clinical applications. It is noteworthy that the efficacy of MSCs is not reliant on their migration into the skin. Working on the immune system, MSCs can inhibit the chemotaxis and infiltration of immune cells to the skin by down-regulating the expression of skin chemokines and chemokine receptors and reducing the inflammatory and pro-fibrotic mediators. ​Furthermore, to reduce levels of oxidative stress, MSCs may improve vascular abnormalities, and enhance the antioxidant defenses through inducible nitric oxide synthase, thioredoxin 1, as well as other mediators. The oxidative stress environment does not weaken MSCs and may even strengthen certain functions. Regarding fibrosis, MSCs primarily target the transforming growth factor-β signaling pathway to inhibit fibroblast activation. Here, miRNAs may play a critical role in ECM remodeling. Clinical studies have demonstrated the safety of these approaches, though outcomes have varied, possibly owing to the heterogeneity of MSCs, the disorders themselves, and other factors. Nevertheless, the research clearly reveals the immense potential of MSCs in treating autoimmune-related fibrotic skin diseases.

**Conclusion:**

The application of MSCs presents a promising approach for treating autoimmune-related fibrotic skin diseases: SSc and Scl-GVHD. Therapies involving MSCs and MSC extracellular vesicles have been found to operate through three primary mechanisms: rebalancing the immune and inflammatory disorders, resisting oxidant stress, and inhibiting overactivated fibrosis (including fibroblast activation and ECM remodeling). However, the effectiveness of these interventions requires further validation through extensive clinical investigations, particularly randomized control trials and phase III/IV clinical trials. Additionally, the hypothetical mechanism underlying these therapies could be elucidated through further research.

## Background

SSc is a multifaceted and multisystem disease, characterized by microvascular damage, innate and adaptive immune dysregulation, and widespread fibrosis affecting the skin and various organs [1, 2]. The majority of hypotheses regarding SSc pathogenesis focus on the interplay between early immunological events and vascular alterations. This interplay results in the emergence of activated fibrogenic fibroblasts, generally recognized as the pivotal effector cells in the disease [3, 4]. Chronic GVHD (cGVHD) is a morbidity and mortality complication disease following allogeneic hematopoietic stem cell transplantation [5], and it shares clinical characteristics akin to autoimmune diseases [6]. Experimental researches support a three-phase pathophysiological model of cGVHD to simplify its intricate pathogenesis: initial inflammation driven and sustained by the innate immune system, with substantial involvement from damaged endothelial cells; chronic inflammation and immune disorders related to Treg cell dysfunction after early uncontrollable inflammation; and scarring and fibrosis in the late stage promoted by dysregulated immunity and aberrant tissue repair [7].The prevailing pathological alterations observed in cGVHD are Scl-GVHD, characterized by a significant rise in collagen deposition, primarily manifesting as lichen planus-like lesions, sclerotic skin manifestations, and poikilodermatous skin changes [8, 9].

Scl-GVHD and SSc share similarities in developmental fibrosis, vascular abnormalities, and innate and adaptive immune response disorders [10, 11]. As a result of the severe skin fibrosis that manifests in both SSc and Scl-GVHD at the late stage, these diseases are classified into the same group of chronic autoimmune connective tissue diseases [12]. Many immune effector cells (T cells, B cells, macrophages, and others) and myofibroblasts participate in the disease process [13–15]. The primary factors that trigger the initiation and progression of skin fibrosis include dysfunctional immune response, oxidative stress caused by hypoxia originating from vascular lesions, and subsequent fibroblast disorders [16, 17]. The significant immune system dysfunction observed in the skin of these two diseases, distinguishing autoimmune-related skin fibrosis from mere skin fibrosis, explain why SSc and Scl-GVHD collectively referred to as autoimmune-related fibrotic skin diseases in this study. In the skin tissue, it has been reported that autoimmune responses facilitated the proliferation and penetration of T cells, particularly T helper (Th)17 and Th2 cells, B cells, monocytes, or macrophages in the SSc and Scl-GVHD [18–20]. This process results in the secretion of enormous immune mediators and pro-fibrotic growth factors, such as interleukin (IL)-4, IL-6, IL-13, IL-17, TGF-β1, and connective tissue growth factors. This coordination promoted the proliferation and differentiation of fibroblasts, leading to the activation of α-smooth muscle actin (α-SMA)^+^ myofibroblasts. Finally, the synthesis of extracellular matrix (ECM), including collagen I (Col-1), and collagen III (Col-3) was enhanced, resulting in fibrosis of both skin tissue and visceral organs [21–23]. In summary, as the key effector cells of fibrosis, fibroblasts are dependently regulated by immune cells in autoimmune-related fibrotic skin diseases to a large extent. Besides, oxidative stress is also a contributing factor to the development of skin fibrosis and is simultaneously modulated by the immune system. Therefore, considering the underlying mechanisms explicated above, treating autoimmune-related skin fibrosis should focus on the pathological complex of immune response—oxidative stress—fibrosis, emphasizing the contribution of the immune system.

**Fig. 1:**
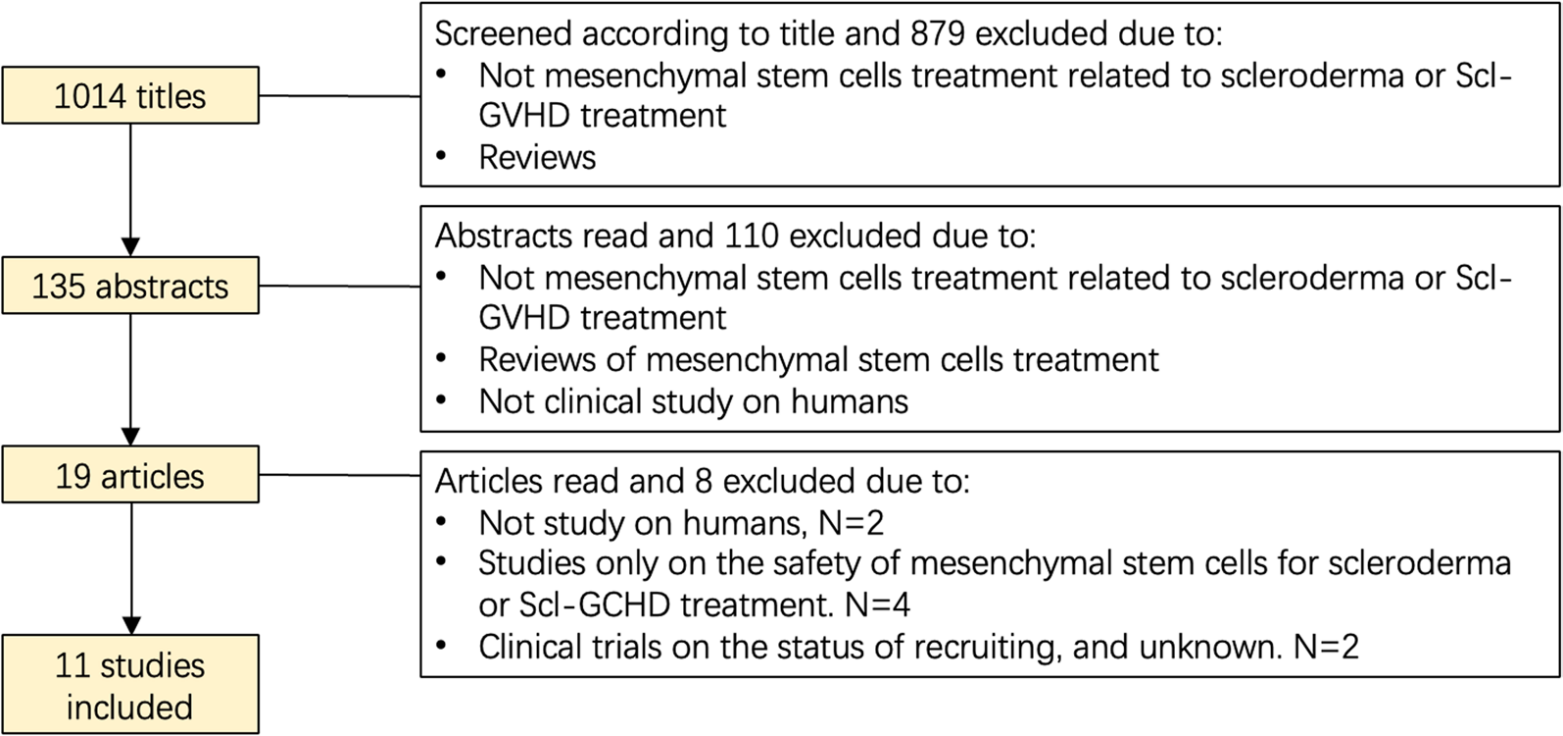
Flowchart of exclusion process ending with 11 included studies

The hypothesis regarding the pathological complex has been corroborated by the inefficacy of anti-fibrotic drugs alone in relation to SSc and Scl-GVHD [24]. The significant dysfunction of the immune system that distinguishes autoimmune-related skin fibrosis from purely fibrotic skin disease is crucial, and emphasis should be placed on treating both fibrosis and the autoimmune aspect. The clinical management of cGVHD is contingent on the severity of the disease. While mild manifestations may suffice with local treatment, moderate and severe forms invariably require systemic immunomodulatory or immunosuppressive therapies [25]. In the case of Scl-GVHD, topical glucocorticoids or calcineurin inhibitors could be additionally used as first-line therapy for drying and flaking skin [26] and ointments and creams are also recommended to relieve symptoms. Limited cutaneous SSc (lSSc) patients are frequently treated with topical corticosteroids and tacrolimus, as well as systemic methotrexate and mycophenolate mofetil [27]. For diffuse cutaneous SSc (dSSc), systematic methotrexate, mycophenolate mofetil, and cyclophosphamide are recommended routine treatments. In addition, immunosuppressive medicine such as rituximab may also be an alternative choice if the clinical therapeutic effect of routine treatments was poor [27]. However, to satisfy the demand for immune regulation and skin fibrosis improvement, traditional immune suppressive medicines may occasionally escalate the incidence rates and mortality rates associated with treatment, especially among steroid-refractory SSc and Scl-GVHD patients [24, 28]. Therefore, it is imperative to develop innovative treatments involving MSCs for autoimmune-related fibrotic skin diseases, encompassing immune regulation, inflammation suppression, oxidation inhibition, and fibrosis constriction [29]. This article presents a compelling rationale for applying MSCs in treating SSc and Scl-GVHD. MSCs have demonstrated the suitability and efficacy in overcoming these diseases in theory. Additionally, numerous clinical studies have exhibited positive long-term results with MSC-based therapies for autoimmune-related fibrotic skin diseases [30]. The diagram of article selection are shown in the Fig. 1 and the full text of 11 studies is retrieved for evaluation. This review aim to summarize the therapeutic effect and theoretical mechanisms of MSC-based therapy in treating autoimmune-related fibrotic skin diseases, SSc and Scl-GVHD, providing novel insights and references for further clinical applications. The discussion will focus on rebalancing immune and inflammatory disorders, enhancing antioxidant defenses, inhibiting overactivated fibrosis (including fibroblast activation and ECM remodeling), and managing the variability of MSCs in clinical and preclinical.

## MSCs alleviate the autoimmune-related skin fibrosis by regulating the immune response

**Fig. 2:**
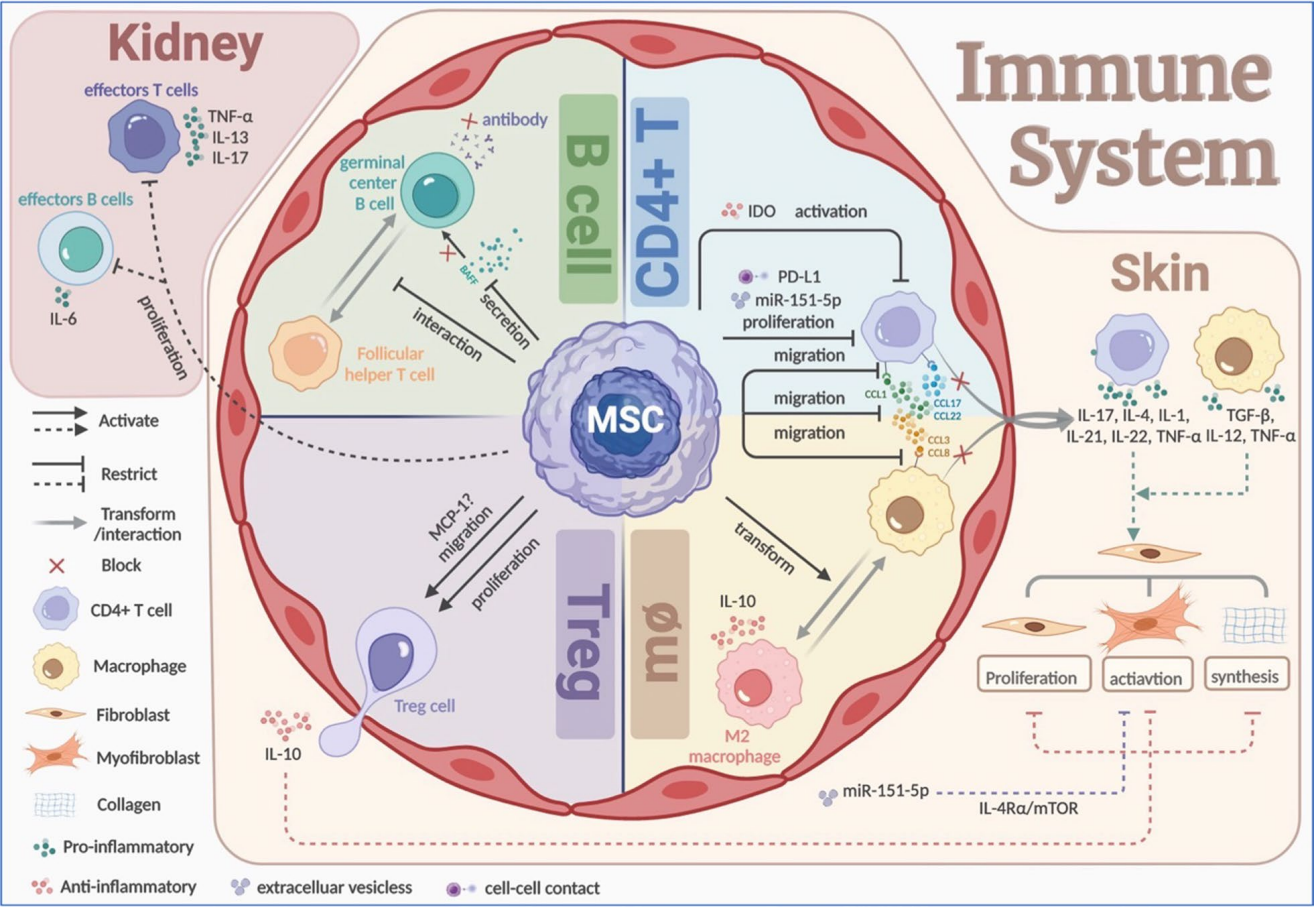
Therapeutic value of MSCs to the pathogenic triad—the immune disorders. In skin, a lot of pro-inflammatory factors (IL-1, IL-4, IL-12, IL-17, IL-21, IL-22, TNF-α, TGF-β) from CD4+ T cells and macrophages can trigger dermal fibrosis. To alleviate inflammation, MSCs inhibit the activation, proliferation, and migration by targeting chemokines and their ligands of CD4+ T cells; inhibit the migration of macrophages while promoting the transformation to anti-inflammatory M2 phenotype; facilitate the migration and proliferation of Treg cells to secrete anti-inflammatory factors IL-10; and inhibit the activation of B cells by reducing BAFF and interaction between germinal center B cells and follicular helper T cells. In the kidney, MSCs inhibit the proliferation of effectors T and B cells. By Biorender (https://biorender.com/). *IL* interleukin, *TNF-α* tumor necrosis factor-α, *TGF-β* transforming growth factor-β, *MSCs* mesenchymal stem cells, *BAFF* B-cell activating factor of the TNF family

MSCs exert a regulatory effect on all participants in the immune system (Fig. 2). Particularly in SSc and Scl-GVHD, MSCs play an essential role in skin homeostasis by inhibiting the proliferation and recruitment of CD4+ T cells and macrophages, while promoting that of suppressor T cell—T regulatory (Treg) cells. Additionally, MSCs suppress the immune response of B cells. Thus, MSCs reduce the pro-inflammatory mediators secreted by CD4+ cells and macrophages, and suppress the expression of autoantibodies by B cells, while they increase anti-inflammatory mediators from Treg cells.

### MSCs influence the migration of T cells and the release of inflammatory factors without needing to migrate into the skin

It is noteworthy that MSCs do not need to migrate into the skin to operate, and MSCs are eliminated in one week after intravenous infusion with a short duration in vivo [13, 31–33]. In contrast, their long-term efficacy is at least 6 weeks in animal models and 12 months in the human body [33, 34]. This effect can even be observed long after elimination [31]. Except for intercellular contact, this phenomenon proves that most MSCs achieve long-term efficacy through paracrine signaling of cytokines, growth factors, and EVs [35]. Not only do MSCs not migrate to the skin, but they also hinder the chemotaxis and infiltration of immune cells to the cutaneous region by down-regulating the expression of cutaneous chemokines and chemokine receptors on immune cells. Among the chemokine ligands (CCL), CCL2, CCL3, and CCL5 are the most critical in fibrosis and inflammation of SSc and Scl-GVHD, serving as vital media for leukocytes to transport [36, 37]. Moreover, CCL17 and CCL22, as the ligands of chemokine receptor (CCR) 4, influence the severity of skin sclerosis and are also integral in recruiting the memory T cells [38, 39]. MSCs can reduce the expression of CCL1, CCL3, CCL8, CCL17, CCL22, and CCR4 on Th2, Th17, as well as Th22 cells, CCR8 on Th2 cells, and CCR1 on CD11β+ macrophages in autoimmune-related fibrotic skin to hinder the arrival of CD4+ T cells, including Th17 and Th2 cells, and CD11β+ macrophages, in particular [13, 40, 41]. Regardless, the infiltration of Th2 may be alleviated by miR-151-5p, a regulatory miRNA from MSC-EVs that can inhibit fibrotic disease [41]. As for CD8+ T cells, their reduction in the skin has been reported since using adipose-derived stem cells (ADSCs) [42]. No down-regulation of CD8+ T cell-related chemokine receptors such as CCR5 has been found. Nonetheless, the decrease of immune effector cells associated with SSc and Scl-GVHD in the skin will ultimately benefit skin fibrosis primarily through a consequent decrease in the secretion of pro-fibrotic factors. It is widely acknowledged that macrophages are a significant source of TGF-β, the most critical trigger for the activation of fibroblasts which leads to ECM over-synthesis in the skin tissue and other organs [43]; T cells excrete a large number of specific cytokines, including IL-17 from Th17 cells, IL-2 from CD4+ T cells, and IL-4 from Th2 cells, as well as IL-1, IL-21, IL-22, tumor necrosis factor-α (TNF-α), and other pro-inflammatory mediators [41, 44]. Besides, Treg cells and their anti-inflammatory secretion such as IL-10 in the skin of cGVHD are instead promoted by MSCs, through a mechanism that cannot be explained by the decrease of CCR4 expression [44]. However, this phenomenon may be linked to the induced up-regulation of monocyte chemotactic protein 1 (MCP-1) toward Treg cells. In other words, MCP-1 from MSCs can recruit T cells and activate FAS pathway-mediated apoptosis of T cells, which transiently leads to high levels of TGF-β from macrophages and brings about the augmentation of Treg cells in mice with SSc [45].

### MSCs suppress the immune response of B cells and impede the production of autoantibodies

Apart from infiltration accommodation, B cell response, including the interaction between follicular Th cells and germinal center B cells, as well as the ratio of B-cell activating factor of the TNF family (BAFF) to B cells, also impacts from the umbilical cord (UC)-MSC-EVs treatment, resulting in improvement of Scl-GVHD model [40]. Therefore, the levels or titer of anti-Scl-70 antibody and serum antinuclear antibody, associated with fibrosis development, are declining in SSc patients [33, 34].

### MSCs regulate the proliferation of immune cells

With regard to immune cell proliferation, MSCs inhibit effectors T and B cells in the spleen, while inhibiting Treg cells until they migrate into the circulatory system [42]. The immunosuppressive effects of MSCs are selectively mediated through cell-to-cell contact of programmed death-ligand 1 (PD-L1) expression, leading to a decrease in both the number and function of mature Th17 cells in the serum [46]; the release of miR-151-5p by MSC-EVs weakens Th2 cell infiltration and reduces their numbers in the serum. However, there is no significant impact observed on the number of Th17 or Th1 cells in the serum. Furthermore, miR-151-5p directly targets IL-4 receptor α (IL-4Rα), thereby interrupting the IL-4Rα/mTOR pathway, which could impede the activation of resting fibroblasts by TGF-β [47].

In brief, MSCs function as a paracrine signaling and EV-based barrier, impeding the arrival of immune cells into the dermis and hindering the inflammatory and fibrotic responses of the immune system. MSCs thereby can attenuate the differentiation, generation, and extracellular matrix production of fibroblasts.

### MSCs secrete anti-inflammatory mediators and possess plasticity and stable immunosuppressive abilities

In addition, MSCs can produce various anti-inflammatory mediators, especially indolemine 2,3-dioxygenase (IDO-2,3), IL-1Rα, tumor necrosis factor α stimulated gene 6 (TSG-6), and prostaglandin E2 (PGE-2) [35, 48]. IDO plays a critical role in inhibiting the tryptophan decomposition in T cells, thereby maintaining the adequate level of signal tryptophan. In the event of tryptophan deficiency, the mTOR pathway is activated, promoting aerobic glycolysis and subsequent T cell activation [49]. MSCs can promote the polarization of monocytes toward anti-inflammatory M2 macrophages, leading to an increase in IL-10 levels while simultaneously decreasing levels of TNF-α and IL-12 [50]. However, these inhibitory phenotypes may shift toward inflammation-supportive ones under certain circumstances, such as exposure to defective immune cells in vitro [51]. This plasticity of MSCs implies that their effects on disease should be explored based on specific pathophysiological backgrounds. Despite the cellular functional impairments of bone marrow-derived (BM) MSCs due to oxidative stress with SSc in vitro, such as cell lifespan, apoptosis, proliferation, and pro-angiogenesis capacity, many studies have repeatedly and consistently demonstrated that both SSc-derived and newly introduced MSCs can still sustain their immunosuppressive potential. This potential is evident in the consistent inhibition of CD4+ and CD8+ T cells and constant induction of functional Treg cells [52–56]. The resilience of MSCs in the unfavorable environment of SSc may be attributed to a higher level of IL-6. Studies have shown that even without IL-1Rα or IL-6, MSCs can still reduce skin thickness and collagen content in hypochlorite (HOCl)-induced SSc mice, indicating that IL-6 is not a crucial factor in the anti-fibrotic effect of healthy MSCs against SSc [57]. However, a higher level of IL-6 from SSc-MSCs can trigger and sustain the inhibition of T cells and induction of Treg cells by MSCs, while camouflaging the aging of MSCs [54].

To further enhance the function of MSCs, it is advisable to prime them with pro-inflammatory factors, such as interferon γ (IFN-γ), TNF-α, IL-1α, IL-1β, IL-17 [58]. This approach can prompt the immunomodulatory response from MSCs in advance, resulting in the up-regulation of anti-inflammatory secretion [IL-10, IDO, cyclooxygenase (COX)-2, PGE-2, TSG-6, CCL12, chemokine (C-X-C Motif) ligand 2 (CXCL2), CCL4], and direct immune cell behaviors [59–66], which can lead to significantly improved therapeutic outcomes for MSCs in treating immune-related disorders in GVHD models [66, 67]. Nevertheless, the immunomodulatory potency of MSCs in clinics varies depending on the pathological background and tissue type, which partially dictates the suitability of tissue-derived MSCs for specific immune-related fibrotic diseases. For instance, in mice with asthma, BM-MSCs may be more effective than ADSCs due to their superior ability to reduce eosinophil infiltration and collagen deposition in the lungs, possibly owing to the increased polarization from monocytes to the M2 phenotype [68]. On the other hand, in mice with SSc, ADSCs are superior to BM-MSCs due to the noteworthy observation that ADSCs reduce the presence of Th17 cells in peripheral blood, which are closely associated with the progression of SSc. Additionally, ADSCs can drain away pro-inflammatory factors, such as TNF-α, IL-1β, IL-6, and IL-17, while simultaneously increasing Treg cells and IL-10 to confront SSc precisely [32].

## MSCs alleviate the autoimmune-related skin fibrosis by resisting oxidant stress

### MSCs improve vascular abnormalities to alleviate oxidative stress

The etiology of oxidative stress in SSc is hypoxia resulting from vascular abnormalities [15]. Despite the apparent tissue hypoxia observed in SSc, there is a lack of evidence supporting compensatory angiogenesis [53]. MSCs can promote angiogenesis and vasculogenesis in SSc by serving as an alternative source of endothelial progenitor cells (EPCs) and pericytes, up-regulating the expression of pro-angiogenic factors, and alleviating oxidative stress [1, 53, 69]. MSCs express specific characteristic proteins of mature endothelial cells such as von Willebrand factor (vWF), vascular endothelial growth factor receptor (VEGFR)-1, VEGFR-2, vascular endothelial (VE)-cadherin, and vascular cell adhesion molecule 1 (VCAM1), which are considered supplementary sources of EPCs [53]. In the pathological microenvironment of SSc, TGF-β and platelet-derived growth factor receptor B (PDGFB) induce differentiation of SSc-MSCs into pericytes. These pericytes exhibit a more mature myofibroblast-like phenotype with high expression levels of α-SMA and smooth muscle 22 α (SM22α). When co-cultured with healthy vascular endothelial cells in vitro, this phenotype can reprogram the cells to promote angiogenesis behavior and improve formation of endothelial cell tubes [1]. In vitro overexpression experiments have demonstrated that SSc-MSCs overexpressing stromal cell-derived factor 1 (SDF-1) and VEGF produce potent pro-angiogenic effects. The blocking antibody experiments have also indicated that these cytokines derived from MSCs are responsible for this strong pro-angiogenic effects. The release of these factors in the local microenvironment can further stimulate their up-regulation and promote redistribution of CXC receptor 4 (CXCR4) and TGF-β receptor II (TGF-βRII) from the cell cytoplasm to the cell surface and focal adhesion contacts, respectively, which facilitate endothelial cell generation in the dermal microvasculature to promote angiogenesis [69].

### MSCs enhance the antioxidant defenses to alleviate oxidative stress

**Fig. 3:**
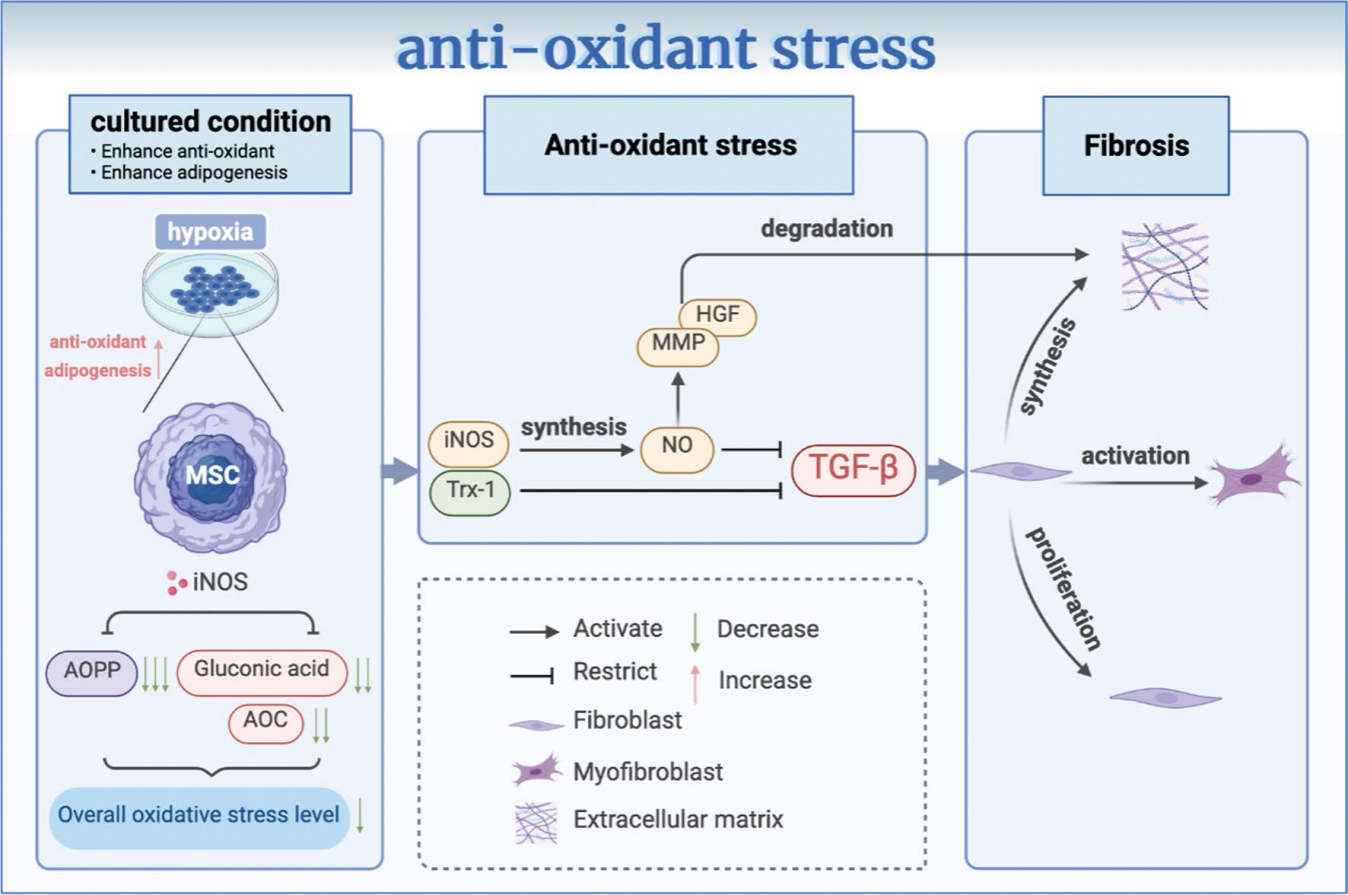
Therapeutic value of MSCs to the pathogenic triad—the oxidant stress. The hypoxia cultured condition enhances the antioxidant and adipogenic capacity of MSCs. Secreted by MSCs, iNOS significantly drops AOPP and neutralize its suppression to gluconic acid and AOC, to regulate the overall oxidative stress level. iNOS helps to the synthesis of NO, increasing MMP and HGF for dermal ECM degradation and restraining TGF-β. MSCs also secrete Trx-1 to weaken TGF-β, which prevent fibroblasts from proliferating, activating and synthesizing ECM. By Biorender (https://biorender.com/). *AOPP* advanced oxidation protein product, *AOC* antioxidant capacity, *ECM* extracellular matrix, *iNOS* Inducible nitric oxide synthase, *MSCs* mesenchymal stem cells, *MMP* matrix metallopeptidase, *HGF* hepatocyte growth factor, *Trx-1* thioredoxin 1, *TGF-β* transforming growth factor-β

MSCs can significantly enhance the antioxidant defenses and reduce levels of oxidative stress in patients with SSc (Fig. 3). Under treatment with MSCs, particularly advanced oxidation protein product (AOPP) levels in serum decreased. However, the levels of manganese superoxide dismutase (SOD) 2, a kind of antioxidant enzyme, and total antioxidant capacity (T-AOC) showed an increase, and these changes were found to be significantly associated with fibrotic flourishing [33]. iNOS is the key anti-fibrotic mechanism associated with MSCs against SSc, particularly in nitric oxide (NO)-related antioxidant stress function. Lacking iNOS in MSCs could not reduce AOPP regularly. Even if MSCs could induce more gluconic acid secretion and enhance the antioxidant capacity (AOC), the overall oxidative stress remained high after carefully considering both AOPP and AOC [70]. MSCs with iNOS can efficiently synthesize NO. Despite being a pro-oxidant in physiological conditions, NO has been proved to be an effective antioxidant in the case of specific pathological conditions, such as SSc, as demonstrated through various preclinical models; MSCs with iNOS can efficiently synthesize NO. Despite being a pro-oxidant in physiological conditions, NO has been proved to be an effective antioxidant in the case of specific pathological conditions such as SSc, as demonstrated through various preclinical models [71]. The presence of NO can impede the TGF-β pathway and the activation of myofibroblasts. At the same time, it promotes matrix metallopeptidase (MMP) and hepatocyte growth factor (HGF), degrades collagen fibers, and promotes ECM remodeling, thereby restraining fibrosis in various organs [72–74]. Nevertheless, whether iNOS exists or not does not significantly impact the regulation of tissue inhibitors of metalloproteinase (TIMP) 1 or the ratio of MMP1/TIMP by MSCs [70]. It is also worth noting that the absence of iNOS does not impair the function of MSCs, such as anti-fibrotic effects that depend on inflammation and immunomodulation. In HOCl mouse models, iNOS^−^ MSCs showed no significant decline in secretion of high levels of inflammatory factors such as IL-1β and IL-6 compared to iNOS^+^ MSCs [70]. Additionally, experiments in vitro have shown that BM-MSCs cultured under hypoxic conditions can resist oxidative stress damage and inhibit the TGF-β pathway through the essential oxidoreductase Trx-1 [75]. Another experiment on hypoxia in mice with SSc suggested that ADSCs exposed to hypoxic conditions would mediate TGF-β, α-SMA, and hydroxyproline levels, resulting in a neater fiber arrangement and a thinner dermal thickness compared to the control group [76]. These findings suggest that the anti-fibrotic effect of MSCs cultured under hypoxic conditions may be enhanced and that the oxidative stress environment of SSc may not weaken MSCs, but rather strengthen their ability to inhibit the TGF-β signaling pathway. Moreover, AOPP, an oxidative stress marker of SSc [77], is a potential predictive indicator for MSC-based treatment of SSc. AOPP level is negatively correlated with the MSC proliferation rate and MSC proliferation rate is inversely correlated with the apoptosis rate. Therefore, the lower the AOPP level, the higher the MSC proliferation rate and the lower the MSC apoptosis rate can be. Besides, the immunosuppressive ability of MSCs remains stable despite exposure to an oxidative stress environment. Therefore, MSC infusion may be a promising therapeutic option for SSc patients with low oxidative stress [52]. Even in severe oxidative stress circumstances, the enhanced antioxidant defense capacity and adipogenic differentiation potential of MSCs may compensate for the shortage of the proliferation rate, thereby maintaining the overall function of MSCs [52]. In SSc mice treated with MSCs, it has been observed that the skin gradually regained its standard thickness, which can be attributed to increased adipogenic differentiation potential and elevated expression of fatty acid synthesis-related genes, both of which are stimulated by the oxidative stress environment in SSc condition [15, 52, 78]. These differentiated adipose cells share similar variability with healthy BM-MSCs [79].

## MSCs alleviate the autoimmune-related skin fibrosis by miRNAs, inhibiting TGF-β pathway and supporting ECM remodeling

**Fig. 4:**
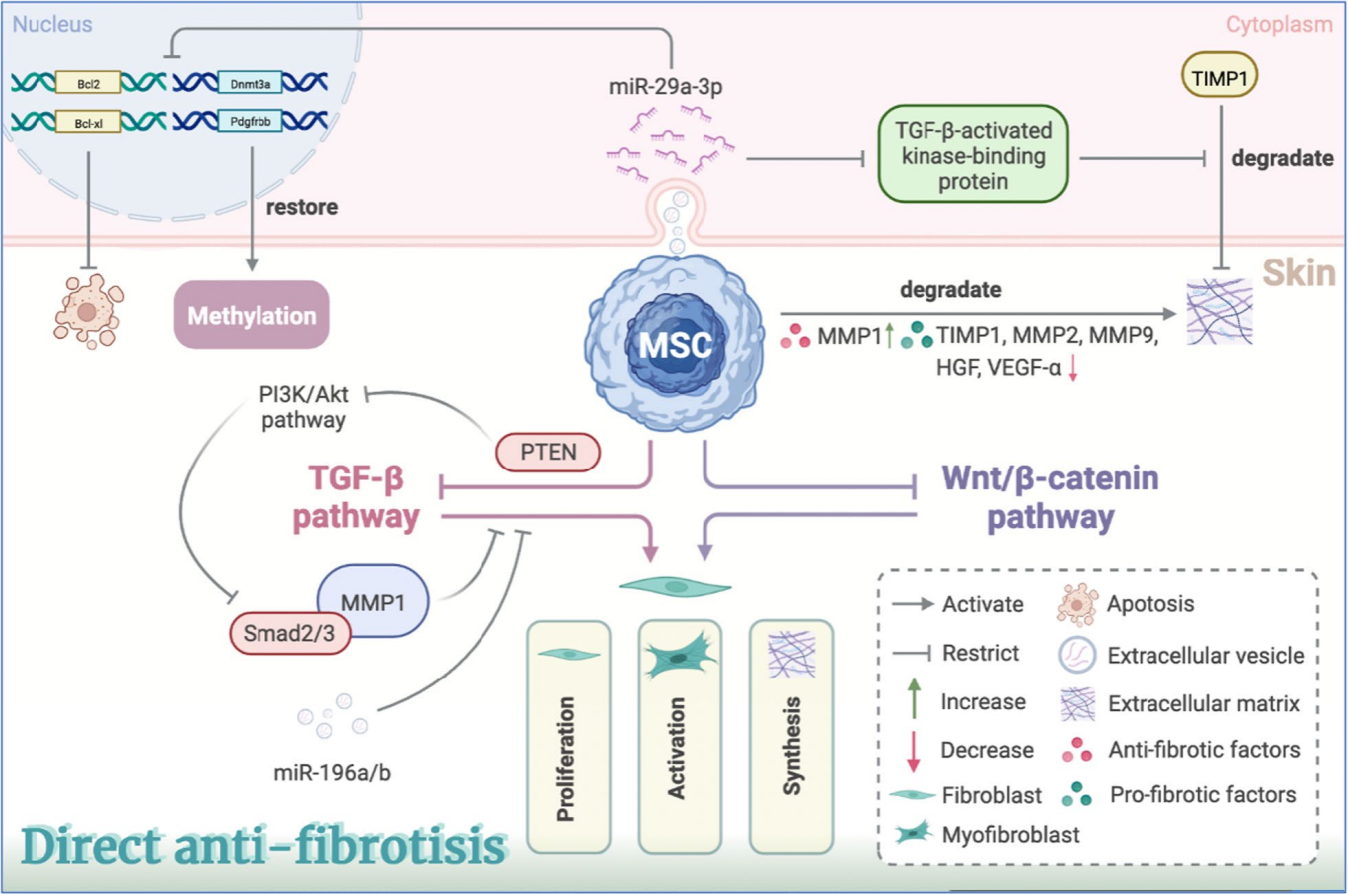
Therapeutic value of MSCs to the pathogenic triad—fibrosis. MSCs release miR-29a-3p to attenuate the expression of anti-apoptotic genes (Bcl2, Bcl-xl), methylation-related genes (Dnmt3α), and PDGFRB, to promote fibroblast apoptosis and restore methylation. MSCs promote ECM degradation through up-regulating anti-fibrotic factors (MMP1) and down-regulating pro-fibrotic factors (TIMP1, MMP2, MMP9, HGF, VEGF-α), or inhibiting the suppression of ECM degradation with miR-29a-3p targeting at TGF-β-activated kinase-binding protein. To regulate fibroblast proliferation, activation, and ECM synthesis, MSCs block the Wnt/β-catenin pathway, and restore the expression of PTEN to restrain the PI3K/Akt-mediated Smad-2/3 and MMP1 superphosphorylation, to inhibit the TGF-β pathway. By Biorender (https://biorender.com/). *Dnmt3α* DNA methytransferase 3α, *PDGFRB* platelet-derived growth factor receptor B, *ECM* extracellular matrix, *MSCs* mesenchymal stem cells, *MMP* matrix metallopeptidase, *TIMP1* tissue inhibitor of metalloproteinases 1, *HGF* hepatocyte growth factor, *VEGF-α* vascular endothelial growth factor α, *TGF-β* transforming growth factor-β, *PTEN* Phosphatase and tensin homolog

After inhibiting immunity and inflammation in the early stages of the disease, MSCs could alleviate skin fibrosis in the late stage by releasing miRNAs, blocking TGF-β signaling and, enhancing tissue remodeling (Fig. 4) [32, 42]. Among these, the inhibition of TGF-β signaling may be linked to phosphatase and tensin homolog (PTEN), a bispecific phosphatase with dual activity in lipid phosphatase and protein phosphatase. MSCs can block PI3K/Akt pathway by restoring PTEN expression, thereby eliminating the TGF-β-mediated superphosphorylation of Smad-2/3 and MMP1 to reduce fibroblast transdifferentiation in the skin of Scl-GVHD mice [13]. Moreover, since the anti-fibrotic effect of MSC-EVs is completely eliminated with the addition of the miR-29a-3p antagonist, miR-29a-3p may also play an essential role in anti-fibrosis [80]. It is acknowledged that miR-29a-3p is only down-regulated in the scleroderma spectrum disorders and has been shown to decrease the expression of anti-apoptosis genes such as B cell lymphoma (Bcl) 2 and Bcl-xl, methylation-related genes like DNA methytransferase 3α (Dnmt3α), and PDGFRB, eventually promoting fibroblast apoptosis and the restoration of abnormal methylation in SSc skin [80, 81]. Besides, miR-196a/b, including miR-196a and miR-196b-5p, which block the TGF-β-mediated synthesis of Col-1α2 in dermal fibroblasts of bleomycin-induced SSc mouse models, may be one of the mechanisms by which MSC-EVs inhibit skin fibrosis in SSc [82, 83]. It is illustrated that there is a more mature myofibroblast phenotype, overexpressing fibrotic markers such as α-SMA, SM22α, and TGFβR-II with BM-MSCs in the SSc compared to healthy individuals [34, 79]. Besides, CD248 overexpression in SSc-MSCs may be involved in TGF-β and PDGFB pathways, promoting pericell–mesenchymal transformation and skin fibrosis [84]. In addition to the mechanism mentioned above in fibroblast activation and proliferation, MSCs and MSC-EVs could interfere with the secretion of some enzymes, growth factors, and other proteins relevant to ECM remodeling, to hinder or reverse skin fibrosis in autoimmune diseases directly. As a member of the miR-29a family, miR-29a-3p aims at down-regulating TGF-β-activated kinase-binding proteins, which act as modulators of TIMP1, thereby intervening in ECM deposition [85, 86]. In HOCl-induced mouse models of SSc, MSCs increase MMP1 while reducing the secretion of TIMP1 and other metalloproteinases such as MMP2 and MMP9, which show distinct biological characteristics from MMP1. This leads to the precipitation of collagen degradation and a reduction in the expression of fibrotic growth factors, HGF and VEGF-α, ultimately weakening the induction of dermal fibroblast collagen synthesis [33].

### The variables of MSCs and interaction of MSCs and immunosuppressants for autoimmune-related fibrotic skin diseases

Regardless of the histocompatibility of MSCs, tissue sources, injection dose, infusion frequency, route of administration, and whether combined with other therapeutic modalities, a meta-analysis has shown that MSC-based therapy can remarkably alleviate cutaneous fibrosis and is generally safe [48, 87]. Nonetheless, there are concerns regarding the variability of MSCs that need to be addressed to stabilize the clinical effects, as their heterogeneity, which mainly includes preclinical variables during the production process of MSCs and clinical variables from the therapeutic course of MSCs, contributes significantly to the mixed clinical outcomes [88]. Regardless of the duration of treatment, the improvement of skin fibrosis after MSCs treatment was considered as the major progress in the majority of clinical and animal studies in Table 1 [10, 30, 32–34, 41, 42, 75, 78, 89–99]. In clinical studies, the improvements of skin fibrosis are primarily manifested as the reducing skin thickness, increasing skin elasticity, and shrinking lesion area [10, 30, 34, 78, 89, 90, 92–96]. Several clinical studies of SSc also reported significant improvements in vascularization, including the restoration of blood vessels in the hands and limbs and the alleviation of Raynaud's phenomenon [89–92]. The improvements of skin fibrosis shown in animal studies primarily include thinner skin thickness, increasing ECM remodeling, and less expression of fibrosis factors [32, 33, 41, 42, 75, 97–99]. Additionally, the restoration of inflammation levels is also an significant feature found in the majority of these studies [10, 32–34, 41, 93, 94, 97, 99].

**Table 1:** Summary of clinical trials and preclinical studies of MSC for autoimmune-related fibrotic skin diseases

Clinical trials								
Condition	Subject	Patients number (study type)	MSC Source and route of Injection	Frequency (time)	Dose (cells/kg)	Main results (follow-up period)	NCT number	References
SSc (Severe diffuse)	Human	1 (Case report)	Allo-BM IV	Single	1 × 10^6^/kg	Reduction of ulceration and pain, improvement of hand vasculopathy	N/A	[91]
SSc	Human	5 (Phase 1)	Allo-BM IV	Single	0.22–1.8 × 10^6^/kg	Improvement of skin fibrosis and vasculopathy (6 M)	N/A	[90]
SSc (limb ischemia)	Human	1 (Case report)	Auto-BM IV	Triple (0, 1, 2 M)	0.8–0.9 × 10^6^/kg	Reduction of necrotic skin areas (0–1 M), epidermal thickness (2 M). Revascularization of extremities (2 M). Increase of angiogenic factors (2 M)	N/A	[92]
SSc	Human	14 (N/A)	Allo-UC + PE IV	Triple PE (1, 3, 5 D) + pulse cyclophosphamide + Single MSC (8 D)	1 × 10^6^/kg	Improvement of mRSS. Reduction of inflammatory markers (12 M)	NCT00962923	[34]
SSc (Localized skin)	Human	6 (Case report)	Auto-AD IH	Single	4–8 × 10^6^	Disease stabilization for all patients and improvement of skin elasticity in 4/6 patients	N/A	[78]
SSc (Finger ulcers)	Human	12 (Phase 1)	Auto-SVF IH	Single	1.4 × 10^6^	Improvement of pain, grasping capacity, finger edema, Raynaud's phenomenon, quality of life	NCT01813279	[89]
SSc (Severe diffuse)	Human	20 (Phase 1/2)	Allo-BM IV	Single	1 × 10^6^/kg, or 3 × 10^6^/kg	Improvement of skin fibrosis (1 Y). Increase of Breg cells (CD24^hi^CD27^pos^CD38^lo/neg^ memory B) and IL-10	NCT02213705	[93, 94]
Scl-GVHD	Human	4 (Case report)	Allo-BM IV	4–8 (1–2 W interval)	1.0–2.0 × 10^7^	Improvement of skin stiffness, pigmentation and mRSS (4.6–23 M). Increase of Th1 cells, IFN-γ, IL-2, and decrease of Th2 cells, IL-10, IL-4 (2 W)	N/A	[10]
Scl-GVHD	Human	1 (Case report)	Allo-UC + rituximab IV	Twice (0, 3 W)	2 × 10^6^/kg	Improvement of skin sclerosis in the abdomen and extremities (3 W). Disappearance of skin sclerosis, pigmentation, and other skin involvement (4 M)	N/A	[121]
cGVHD	Human	10 (Phase 1)	Allo-BM IV	Single	1 × 10^6^/kg	Improved skin response in 1/5 patients with clinical improvement (2 W)	NCT01318330	[30]
cGVHD	Human	9 (Phase 2)	Allo-BM IV	≥ 6 (4–6 W interval)	2 × 10^6^/kg	Improved quality of life, and reduced requirements of traditional immunosuppressive drugs (12 M). Almost complete disappearance of epidermal lesions in 6/9 patients (34–99 M)	NCT01522716	[95]

Animal Model	Subject	MSC source and route of Injection	Frequency (time)	Dose	Main results	Mechanisms	References
Bleomycin-induced skin fibrosis mouse	Animal	syngeneic BM IH	Daily (4 W)	1 × 10^6^	Improvement remodeling matrix responsible for normal collagen arrangement in skin. Reduction of inflammation and α-SMA^+^ myofibroblasts	Down-regulation of TGF-β, collagen I and HSP47 expression	[97]
HOCl-induced SSc mouse	Animal	Syngeneic/Allo-BM, or human ASCs & BM-MSCs IV	Single or twice (0, 21 D)	2.5 × 10^5^	Reduction of fibrotic, inflammatory and oxidative markers in skin & lungs. Improvement of matrix remodeling	N/A	[33]
Type 1 tight skin (Tsk1) mouse	Animal	Allo-BM IV	8 W	1 × 10^5^/kg	Improvement of osteopenia	Down-regulation of the IL-4R pathway by miR-151-5p in MSC-EV	[122]
Bleomycin-induced skin fibrosis mouse	Animal	Trx-1-overexpressing BM-MSCs IH	Daily (3 W)	1 × 10^6^	Reduction of skin fibrosis and apoptosis, promotion of BM-MSC survival and differentiation into endothelial cells	TRX1-mediated inhibition of oxidative stress	[75]
Bleomycin-induced skin sclerosis mouse	Animal	human micro-fat + SVF IH	Single	0.5 ml micro-fat + 0.0114 ml SVF	Decrease in the established dermal fibrosis and increase in local vascularization	SVF cell pro-angiogenic properties, and the favorable conditions for grafting and survival offered by micro-fat	[98]
Bleomycin-induced skin sclerosis mouse	Animal	Human ADSC-assisted lipotransfer IH	Single	0.3 ml fat + 1 × 10^6^ ADSCs	Improvement of the skin texture and collagen content. Reduced TGF-β1 and collagen III expression	N/A	[99]
Bleomycin-induced skin fibrosis mouse	Animal	Auto-AD IH	Single	2 × 10^6^	Amelioration of dermal fibrosis Reduction of the skin thickness and the total content of hydroxyproline	Down-regulation of TGF-β1 and up-regulation of VEGF	[41]
Bleomycin-induced scleroderma and Scl-GVHD mouse	Animal	Allo-AD IV	Single	2 × 10^5^	Attenuation of skin fibrosis	Suppression of infiltration of CD4+, CD8+ T cells and macrophages into the dermis. Reduction of the messenger RNA expression of collagen and pro-fibrotic cytokines in the skin	[42]
Bleomycin-induced skin fibrosis mouse	Animal	Allo-AD IH	Single	2.5 × 10^4^	Provision of dendritic cell-derived signals improved survival and effectiveness of therapeutically delivered ADSCs	LTβR-mediated activation of ADSCs in the late stage of fibrosis development	[123]
HOCl-induced diffuse SSc mouse	Animal	healthy murine and human ADSCs IV	Single	2.5 × 10^5^	Decrease in skin fibrotic and pro-inflammatory markers	N/A	[32]

*MSC* Mesenchymal stem cell, *Ref.* reference, *SSc* systemic sclerosis, *Scl-GVHD* sclerodermatous graft-versus-host disease, *Allo* allogeneic, *Auto* autologous, *BM* bone marrow, *AD* adipose-derived, *UC* umbilical cord, *SVF* stromal vascular fraction, *IV* intravenous injection, *IH* hypodermic injection, *mRSS* modified Rodnan skin score, *PE* plasmapheresis, *N/A* not applicable, *IL* interleukin, *Th* T helper, *IFN-γ* interferon γ, *TGF-β* transforming growth factor-β, *α-SMA* α-smooth muscle actin, *HSP47* heat-shock protein 47, *IL-4R* IL-4 receptor, *MSC-EV* MSC extracellular vesicle, *Trx-1* thioredoxin 1, *VEGF* vascular endothelial growth factor, *LTβR* lymphotoxin B receptor, *HOCl* hypochlorite, *M* month, *W* week, *D* day

### The preclinical variables of MSCs

Heterogeneity and plasticity of MSCs arising from differences in production and processing include the source, culture conditions, and the number of passages [40, 100, 101]. Regarding the source of MSCs, ADSCs exhibit a more potent immunosuppressive ability for the maturation and differentiation of T cells, B cells, and monocytes than BM-MSCs, while ADSCs demonstrate high proliferation rates in vivo and in vitro [102–109]. ADSCs also possess a more substantial potential for adipogenesis differentiation that is not easily weakened or lost along with increasing passages and age of donor [24], possibly providing an advantage in restoring the subcutaneous fat layer of SSc simultaneously. A descriptive study by Maria et al. comparing the efficacy of human BM-MSCs with human ADSCs in the HOCl-induced SSc mouse model supported that ADSCs were significantly more efficient in alleviating skin fibrosis than BM-MSCs [32]. A total of 18 patients in two SSc clinical trials treated with ADSCs revealed significant improvements in skin elasticity, pigmentation, local skin lesion progression, subcutaneous fat thickness and function, edema, and hand movement [78, 89]. MSCs can be divided into two categories based on the donor source: healthy-originated allogeneic MSC (h-MSC) and SSc patient-originated autologous MSC (SSc-MSC). Compared to h-BM-MSCs, SSc-BM-MSCs have different phenotypes [1, 34]. Although immunosuppressive and hematopoietic functions of SSc-BM-MSCs avoid hefty clout, their cell lifespan and functional activities of proliferation, angiogenesis, and anti-fibrosis are inevitably impaired [53, 55, 56]. The same issue also applies to SSc-ADSCs. SSc does not modify the phenotype, morphology, differentiation, and adhesion capacity of autologous ADSCs. However, it weakens their proliferation rate, metabolic activity, migration, and invasion capacity [78, 110]. The miRNAs expression profile of both SSc-BM-MSCs and SSc-ADSCs has been found in the up-regulation of pathways, including senescence mechanisms and pro-fibrotic behaviors, and the down-regulation of pathways related to cell survival [111]. Recent clinical trials with h-MSCs have demonstrated that they could be an exciting alternative for treating SSc and Scl-GVHD [34, 90, 91]. Both autologous MSCs and allogeneic MSCs are considered to alleviate skin fibrosis in SSc clinically, but which one would be preferred still need further information [42, 112]. Additionally, the optimal culture conditions for MSCs are crucial for proliferation and functional properties. MSCs cultured in acidosis indicated that they produce more anti-inflammatory extracellular vesicles, leading to a more noticeable promotion effect on Treg cells than MSCs in standard conditions [113].

### The clinical variables of MSCs

Meanwhile, proper prescription of injection dose, infusion frequency, administration time, and route of administration is also conducive to the therapeutic efficacy of MSCs and MSC-EVs in autoimmune-related fibrotic skin diseases [40], as evidenced by the dose and frequency dependence of MSC efficacy demonstrated in both preclinical and clinical studies [33, 114]. Currently, the most common injection dose for treating SSc and Scl-GVHD with MSCs is 1 × 10^6^/kg [4, 30, 34, 91]. However, it is worth noting that the relationship between doses and efficacy is not always linear, and the effective dosage range of MSCs should be determined based on clinical application [115]. An animal study has shown that the efficacy of MSC for skin fibrosis is inversely proportional to the dosage administered. In a study conducted on HOCl-induced SSc mice using three different doses of MSC (2.5 × 10^5^, 5 × 10^5^, and 1 × 10^6^), MSCs administered at a dosage of 2.5 × 10^5^ showed the most potent anti-fibrotic effects [33]. On the contrary, there are some significant issues in some research concerning MSCs, where the principal mechanism of anti-fibrosis, immunosuppressive potential, is affected by the dose. Such investigations indicated that the higher the dosage, the stronger the ability of MSCs to inhibit the proliferation, activation, and maturity of T and B cells [52, 116, 117]. The immune efficacy of MSCs will disappear if the infusion is below 10^4^ [116]. Further research is necessary to establish a more precise dose gradient that can account for the formation of this apparent contradiction. Intravenous injection (IV) and hypodermic injection (IH) are the two most common routes for SSc administration. Considering the increased workload of managing extensive skin lesions of SSc and Scl-GVHD, IV is the main route compared to IH. Nevertheless, it is worth noting that MSCs tend to drain into lymphatic organs rather than entering the target organs such as skin through IV [118]. In contrast, IH can effectively reach skin lesions and has the potential to prolong the survival of MSCs. Consequently, IH may be an excellent alternative for lSSc, which involves relatively localized skin lesions and without the excessive difficulty of operation at the same time. Therefore, IV may be more suitable for dSSc, where IH is more challenging to administer. Additionally, early administration and multiple doses are recommended for a long-lasting outcome and the long-term success rate of MSC therapy. Early injection of MSC after allogeneic hematopoietic stem cell transplantation can reduce the severity of Scl-GVHD in several ways, manifested as decreasing skin scores, histopathological scores, skin thickness, skin fibrosis, as well as the mRNA expression level of Col-1 and Col-3 [13]. Since neither SSc nor Scl-GVHD is a rapidly curable disease and the efficacy of MSCs is not once and for all, repetitive intermittent infusions are required to maintain the therapeutic efficacy. In HOCl-induced mouse models of SSc, a single infusion of MSC on day 0 can restrain the progression within one week. However, reinfusion on day 21 can further consolidate and prolong the inhibition of skin thickening and fibrosis markers expression [33]. Likewise, early and multiple interventions have been shown to yield better long-term effects in most cases, including models of heart, liver fibrosis, and HOCl-induced pulmonary fibrosis [88]. Therefore, the administration intervals for Scl-GVHD are typically fixed at 1–2 weeks [10], with a recommended total duration of at least one year [119, 120].

### The interaction between MSCs and immunosuppressants

The combination of MSC and immunosuppressants in the treatment of autoimmune-related fibrotic skin diseases has shown positive trends in several clinical studies [124]. In vitro studies or animal experiments have also demonstrated potential beneficial interactions between MSCs and immunosuppressants [125, 126]. MSCs mitigate the adverse effects of immunosuppressive drugs on T cell subsets [126–128], promoting the expression of anti-inflammatory Treg cells and inhibiting the expression of pro-inflammatory T cell subsets [129]. AD-MSCs enhance the immunosuppressive effect of cyclosporine A on T cells through the inhibition of jagged-1-mediated NF-kB signal [129] MSCs can counteract the up-regulatory impact of immunosuppressants, such as MMF and dexamethasone, on pro-inflammatory T cell subsets such as Th-17 cells. They can even attenuate the expression of inflammatory factors like IL-17. MSCs significantly enhance the down-regulatory impacts of immunosuppressants on IL-4 while collaborating with cyclosporine A and glucocorticoids to diminish the quantity of T-bet-positive cells [127]. Although immunosuppressants interfere with MSCs' function (proliferation, migration, etc.) [130–134], they barely affect the immunosuppressive properties and even prolong the duration of MSCs in vivo [126, 135, 136]. Both cyclosporine and dexamethasone are reported to enhance the promoting effect of MSC on Treg cell expression [126]. In addition, cyclophosphamide can improve the survival rate of MSCs [126], while dexamethasone extends the retention period of MSCs in vivo and mitigates apoptosis of MSCs [137–140]. Although further validations are required, the combination therapy involving MSCs still represents a promising method to reduce immunosuppressant doses while maintaining or potentially enhancing treatment outcomes [126, 127].

## Conclusions

It has become increasingly evident that MSCs and MSC-EVs are advantageous in treating autoimmune-related fibrotic skin diseases (SSc and Scl-GVHD). These therapies have shown particular promise in three main areas: (1) rebalancing immune and inflammatory disorders, (2) enhancing antioxidant defenses, and (3) inhibiting overactivated fibrosis. The safety of MSC-based therapy in clinics has been proved, and it is considered as a potentially effective option for treating SSc and Scl-GVHD. However, several questions remain unresolved. More evidence is needed to compare effectiveness of h-MSCs and SSc-MSCs, as well as MSCs combination therapy and immunosuppressant therapy alone. Further investigations are required to determine the optimal therapeutic dosage range and frequency and duration of infusions for individual patients. Another question, might be addressed by studying skin lesions, is whether generalized IV or localized IH is a more suitable treatment for the disease. Finally, randomized controlled trials that systematically assess the correlation between in vitro and in vivo mechanisms, may provide more definitive evidence on theoretical mechanisms underlying the efficacy of MSC-based therapy.

## Data Availability

Not applicable.

## Abbreviations

SSc: Systemic sclerosis
Scl-GVHD: Sclerodermatous graft-versus-host disease
MSCs: Mesenchymal stem cells
iNOS: Inducible nitric oxide synthase
Trx-1: Thioredoxin 1
TGF-β: Transforming growth factor-β
EV: Extracellular vesicle
cGVHD: Chronic GVHD
Th: T helper
IL: Interleukin
α-SMA: α-Smooth muscle actin
ECM: Extracellular matrix
Col-1: Collagen I
Col-3: Collagen III
Treg: T regulatory
CCL: Chemokine ligand
CCR: Chemokine receptor
ADSCs: Adipose-derived stem cells
TNF-α: Tumor necrosis factor-α
MCP-1: Monocyte chemotactic protein 1
BAFF: B-cell activating factor of the TNF family
UC: Umbilical cord
PD-L1: Programmed death-ligand 1
IL-4Rα: IL-4 receptor α
IDO-2,3: Indolemine 2,3-dioxygenase
TSG-6: Tumor necrosis factor α stimulated gene 6
PGE-2: Prostaglandin E2
BM: Bone marrow-derived
HOCl: Hypochlorite
IFN-γ: Interferon γ
COX: Cyclooxygenase
CXCL2: Chemokine (C-X-C Motif) ligand 2
EPCs: Endothelial progenitor cells
vWF: von Willebrand factor
VEGFR: Vascular endothelial growth factor receptor
VE: Vascular endothelial
VCAM1: Vascular cell adhesion molecule 1
PDGFRB: Platelet-derived growth factor receptor B
SM22α: Smooth muscle 22 α
SDF-1: Stromal cell-derived factor 1
CXCR4: CXC receptor 4
TGFβR-II: TGF-β receptor II
AOPP: Advanced oxidation protein product
SOD: Superoxide dismutase
T-AOC: Total antioxidant capacity
NO: Nitric oxide
AOC: Antioxidant capacity
MMP: Matrix metallopeptidase
HGF: Hepatocyte growth factor
TIMP: Tissue inhibitors of metalloproteinase
PTEN: Phosphatase and tensin homolog
Bcl: B cell lymphoma
Dnmt3α: DNA methytransferase 3α
h-MSC: Healthy-originated allogeneic MSC
SSc-MSC: SSc patient-originated autologous MSC
IV: Intravenous injection
IH: Hypodermic injection
lSSc: Limited cutaneous SSc
dSSc: Diffuse cutaneous SSc
Ref.: Reference
Allo: Allogeneic
Auto: Autologous
AD: Adipose-derived
SVF: Stromal vascular fraction
mRSS: Modified Rodnan skin score
PE: Plasmapheresis
N/A: Not applicable
HSP47: Heat-shock protein 47
LTβR: Lymphotoxin B receptor
M: Month
W: Week
D: Day

## References

[1] Cipriani P, Marrelli A, Benedetto PD, et al. Scleroderma Mesenchymal Stem Cells display a different phenotype from healthy controls; implications for regenerative medicine. Angiogenesis. 2013;16:595–607. 10.1007/s10456-013-9338-9 23413114

[2] Cipriani P, Benedetto PD, Ruscitti P, et al. Impaired endothelium-mesenchymal stem cells cross-talk in systemic sclerosis: a link between vascular and fibrotic features. Arthritis Res Ther. 2014;16:442. 10.1186/s13075-014-0442-z PMC4206764 25248297

[3] Denton CP, Black CM, Korn JH (1996). Systemic sclerosis: current pathogenetic concepts and future prospects for targeted therapy. The Lancet.

[4] Denton CP, Khanna D (2017). Systemic sclerosis. The Lancet.

[5] Baird K, Pavletic SZ (2006). Chronic graft versus host disease. Curr Opin Hematol.

[6] Zeiser R, Blazar BR (2017). Pathophysiology of chronic graft-versus-host disease and therapeutic targets. N Engl J Med.

[7] Cooke KR, Luznik L, Sarantopoulos S (2017). The biology of chronic graft-versus-host disease: a task force report from the national institutes of health consensus development project on criteria for clinical trials in chronic graft-versus-host disease. Biol Blood Marrow Transplant J Am Soc Blood Marrow Transplant.

[8] Cho B-S, Min C-K, Eom K-S (2009). Feasibility of NIH consensus criteria for chronic graft-versus-host disease. Leukemia.

[9] Filipovich AH, Weisdorf D, Pavletic S (2005). National Institutes of Health consensus development project on criteria for clinical trials in chronic graft-versus-host disease: I. Diagnosis and staging working group report. Biol Blood Marrow Transplant J Am Soc Blood Marrow Transplant.

[10] Zhou H, Guo M, Bian C (2010). Efficacy of bone marrow-derived mesenchymal stem cells in the treatment of sclerodermatous chronic graft-versus-host disease: clinical report. Biol Blood Marrow Transplant J Am Soc Blood Marrow Transplant.

[11] Farge D, Loisel S, Lansiaux P, et al. Mesenchymal stromal cells for systemic sclerosis treatment. Autoimmun Rev. 2021;20:102755. 10.1016/j.autrev.2021.102755 33476823

[12] Vanneaux V, Farge-Bancel D, Lecourt S, et al. Expression of transforming growth factor β receptor II in mesenchymal stem cells from systemic sclerosis patients. BMJ Open. 2013;3:e001890. 10.1136/bmjopen-2012-001890 PMC3549232 23299111

[13] Lim J-Y, Ryu D-B, Lee S-E, et al. Mesenchymal stem cells (MSCs) attenuate cutaneous sclerodermatous graft-versus-host disease (Scl-GVHD) through inhibition of immune cell infiltration in a mouse model. J Investig Dermatol. 2017;137:1895–904. 10.1016/j.jid.2017.02.986 28526296

[14] Gur C, Wang S-Y, Sheban F (2022). LGR5 expressing skin fibroblasts define a major cellular hub perturbed in scleroderma. Cell.

[15] Jin J, Ou Q, Wang Z, et al. BMSC-derived extracellular vesicles intervened the pathogenic changes of scleroderma in mice through miRNAs. Stem Cell Res Ther. 2021;12:327. 10.1186/s13287-021-02400-y PMC8179710 34090522

[16] Allanore Y, Simms R, Distler O (2015). Systemic sclerosis. Nat Rev Dis Primer.

[17] Gabrielli A, Avvedimento EV, Krieg T (2009). Scleroderma. N Engl J Med.

[18] Sato S, Hasegawa M, Takehara K (2001). Serum levels of interleukin-6 and interleukin-10 correlate with total skin thickness score in patients with systemic sclerosis. J Dermatol Sci.

[19] Matsushita T, Hasegawa M, Hamaguchi Y (2006). Longitudinal analysis of serum cytokine concentrations in systemic sclerosis: association of interleukin 12 elevation with spontaneous regression of skin sclerosis. J Rheumatol.

[20] O’Reilly S. MicroRNAs in fibrosis: opportunities and challenges. Arthritis Res Ther. 2016;18:11. 10.1186/s13075-016-0929-x PMC4718015 26762516

[21] Huang J, Maier C, Zhang Y (2017). Nintedanib inhibits macrophage activation and ameliorates vascular and fibrotic manifestations in the Fra2 mouse model of systemic sclerosis. Ann Rheum Dis.

[22] Higashi-Kuwata N, Jinnin M, Makino T (2010). Characterization of monocyte/macrophage subsets in the skin and peripheral blood derived from patients with systemic sclerosis. Arthritis Res Ther.

[23] Varga J, Abraham D (2007). Systemic sclerosis: a prototypic multisystem fibrotic disorder. J Clin Investig.

[24] Zhuang X, Hu X, Zhang S, et al. Mesenchymal stem cell-based therapy as a new approach for the treatment of systemic sclerosis. Clin Rev Allergy Immunol. 2022;64:284–320. 10.1007/s12016-021-08892-z 35031958

[25] Link-Rachner CS, Sockel K, Schuetz C (2022). Established and emerging treatments of skin GvHD. Front Immunol.

[26] Penack O, Marchetti M, Ruutu T (2020). Prophylaxis and management of graft versus host disease after stem-cell transplantation for haematological malignancies: updated consensus recommendations of the European Society for Blood and Marrow Transplantation. Lancet Haematol.

[27] Knobler R, Moinzadeh P, Hunzelmann N (2017). European Dermatology Forum S1-guideline on the diagnosis and treatment of sclerosing diseases of the skin, Part 1: localized scleroderma, systemic sclerosis and overlap syndromes. J Eur Acad Dermatol Venereol JEADV.

[28] Fallet B, Walker UA. Current immunosuppressive and antifibrotic therapies of systemic sclerosis and emerging therapeutic strategies. Expert Rev Clin Pharmacol. 2020;13:1203–18. 10.1080/17512433.2020.1832466 33008265

[29] Spees JL, Lee RH, Gregory CA (2016). Mechanisms of mesenchymal stem/stromal cell function. Stem Cell Res Ther.

[30] Yi HG, Yahng S-A, Kim I (2016). Allogeneic clonal mesenchymal stem cell therapy for refractory graft-versus-host disease to standard treatment: a phase I study. Korean J Physiol Pharmacol.

[31] Nauta AJ, Westerhuis G, Kruisselbrink AB, et al. Donor-derived mesenchymal stem cells are immunogenic in an allogeneic host and stimulate donor graft rejection in a nonmyeloablative setting. Blood. 2006;108:2114–20. 10.1182/blood-2005-11-011650 PMC1895546 16690970

[32] Maria ATJ, Toupet K, Maumus M, et al. Human adipose mesenchymal stem cells as potent anti-fibrosis therapy for systemic sclerosis. J Autoimmun. 2016;70:31–9. 10.1016/j.jaut.2016.03.013 27052182

[33] Maria ATJ, Toupet K, Bony C, et al. Antifibrotic, antioxidant, and immunomodulatory effects of mesenchymal stem cells in HOCl-induced systemic sclerosis. Arthritis Rheum. 2016;68:1013–25. 10.1002/art.39477 26474311

[34] Zhang H, Liang J, Tang X, et al. Sustained benefit from combined plasmapheresis and allogeneic mesenchymal stem cells transplantation therapy in systemic sclerosis. Arthritis Res Ther. 2017;19:165. 10.1186/s13075-017-1373-2 PMC5518166 28724445

[35] Maumus M, Jorgensen C, Noël D (2013). Mesenchymal stem cells in regenerative medicine applied to rheumatic diseases: role of secretome and exosomes. Biochimie.

[36] Hasegawa M, Sato S (2008). The roles of chemokines in leukocyte recruitment and fibrosis in systemic sclerosis. Front Biosci J Virtual Libr.

[37] Zhang Y, McCormick LL, Desai SR (1950). Murine sclerodermatous graft-versus-host disease, a model for human scleroderma: cutaneous cytokines, chemokines, and immune cell activation. J Immunol Baltim Md.

[38] Fujii H, Shimada Y, Hasegawa M (2004). Serum levels of a Th1 chemoattractant IP-10 and Th2 chemoattractants, TARC and MDC, are elevated in patients with systemic sclerosis. J Dermatol Sci.

[39] Kusumoto M, Xu B, Shi M (2007). Expression of chemokine receptor CCR4 and its ligands (CCL17 and CCL22) in murine contact hypersensitivity. J Interferon Cytokine Res.

[40] Guo L, Lai P, Lai P, et al. Extracellular vesicles derived from mesenchymal stem cells prevent skin fibrosis in the cGVHD mouse model by suppressing the activation of macrophages and B cells immune response. Int Immunopharmacol. 2020;84:106541. 10.1016/j.intimp.2020.106541 32402950

[41] Chen W, Xia Z-K, Zhang M-H, et al. Adipose tissue-derived stem cells ameliorates dermal fibrosis in a mouse model of scleroderma. Asian Pac J Trop Med. 2017;10:52–6. 10.1016/j.apjtm.2016.10.005 28107865

[42] Okamura A, Matsushita T, Komuro A, et al. Adipose‐derived stromal/stem cells successfully attenuate the fibrosis of scleroderma mouse models. Int J Rheum Dis. 2020;23:216–25. 10.1111/1756-185X.13764 31808305

[43] Ho YY, Lagares D, Tager AM (2014). Fibrosis: a lethal component of systemic sclerosis. Nat Rev Rheumatol.

[44] Lai P, Chen X, Guo L, et al. A potent immunomodulatory role of exosomes derived from mesenchymal stromal cells in preventing cGVHD. J Hematol OncolJ Hematol Oncol. 2018;11:135. 10.1186/s13045-018-0680-7 PMC6286548 30526632

[45] Akiyama K, Chen C, Wang D (2012). Mesenchymal-stem-cell-induced immunoregulation involves FAS-ligand-/FAS-mediated T cell apoptosis. Cell Stem Cell.

[46] Luz-Crawford P, Noël D, Fernandez X, et al. Mesenchymal stem cells repress Th17 molecular program through the PD-1 pathway. PLoS ONE. 2012;7:e45272. 10.1371/journal.pone.0045272 PMC3444478 23028899

[47] Chakraborty D, Šumová B, Mallano T (2017). Activation of STAT3 integrates common profibrotic pathways to promote fibroblast activation and tissue fibrosis. Nat Commun.

[48] Dunn CM, Kameishi S, Grainger DW (2021). Strategies to address mesenchymal stem/stromal cell heterogeneity in immunomodulatory profiles to improve cell-based therapies. Acta Biomater.

[49] Böttcher M, Hofmann AD, Bruns H (2016). Mesenchymal stromal cells disrupt mTOR-signaling and aerobic glycolysis during T-cell activation. Stem Cells Dayt Ohio.

[50] Ma OK-F, Chan KH (2016). Immunomodulation by mesenchymal stem cells: Interplay between mesenchymal stem cells and regulatory lymphocytes. World J Stem Cells.

[51] Le Burel S, Thepenier C, Boutin L (2017). Effect of mesenchymal stromal cells on T cells in a septic context: immunosuppression or immunostimulation?. Stem Cells Dev.

[52] Fonteneau G, Fonteneau G, Bony C, et al. Serum-mediated oxidative stress from systemic sclerosis patients affects mesenchymal stem cell function. Front Immunol. 2017;8:988. 10.3389/fimmu.2017.00988 PMC5585199 28919892

[53] Cipriani P, Guiducci S, Miniati I, et al. Impairment of endothelial cell differentiation from bone marrow-derived mesenchymal stem cells: new insight into the pathogenesis of systemic sclerosis. Arthritis Rheum. 2007;56:1994–2004. 10.1002/art.22698 17530639

[54] Cipriani P, Benedetto PD, Liakouli V, et al. Mesenchymal stem cells (MSCs) from scleroderma patients (SSc) preserve their immunomodulatory properties although senescent and normally induce T regulatory cells (Tregs) with a functional phenotype: implications for cellular-based therapy. Clin Exp Immunol. 2013;173:195–206. 10.1111/cei.12111 PMC3722920 23607751

[55] Larghero J, Farge D, Braccini A, et al. Phenotypical and functional characteristics of in vitro expanded bone marrow mesenchymal stem cells from patients with systemic sclerosis. Ann Rheum Dis. 2007;67:443–9. 10.1136/ard.2007.071233 17526552

[56] Bocelli-Tyndall C, Bracci L, Spagnoli GC, et al. Bone marrow mesenchymal stromal cells (BM-MSCs) from healthy donors and auto-immune disease patients reduce the proliferation of autologous- and allogeneic-stimulated lymphocytes in vitro. Rheumatology. 2007;46:403–8. 10.1093/rheumatology/kel267 16920750

[57] Maria ATJ, Toupet K, Maumus M, et al. Fibrosis development in HOCl-induced systemic sclerosis: a multistage process hampered by mesenchymal stem cells. Front Immunol. 2018;9:2571. 10.3389/fimmu.2018.02571 PMC6230680 30455706

[58] Noronha NDC, Mizukami A, Caliári-Oliveira C (2019). Priming approaches to improve the efficacy of mesenchymal stromal cell-based therapies. Stem Cell Res Ther.

[59] Boregowda SV, Krishnappa V, Haga CL (2016). A clinical indications prediction scale based on TWIST1 for human mesenchymal stem cells. EBioMedicine.

[60] Giri J, Das R, Nylen E (2020). CCL2 and CXCL12 derived from mesenchymal stromal cells cooperatively polarize IL-10+ tissue macrophages to mitigate gut injury. Cell Rep.

[61] English K, Ryan JM, Tobin L (2009). Cell contact, prostaglandin E(2) and transforming growth factor beta 1 play non-redundant roles in human mesenchymal stem cell induction of CD4+CD25(High) forkhead box P3+ regulatory T cells. Clin Exp Immunol.

[62] Galipeau J, Krampera M, Barrett J (2016). International Society for Cellular Therapy perspective on immune functional assays for mesenchymal stromal cells as potency release criterion for advanced phase clinical trials. Cytotherapy.

[63] English K, Barry FP, Field-Corbett CP (2007). IFN-gamma and TNF-alpha differentially regulate immunomodulation by murine mesenchymal stem cells. Immunol Lett.

[64] Wobma HM, Tamargo MA, Goeta S (2018). The influence of hypoxia and IFN-γ on the proteome and metabolome of therapeutic mesenchymal stem cells. Biomaterials.

[65] Krampera M, Cosmi L, Angeli R (2006). Role for interferon-γ in the immunomodulatory activity of human bone marrow mesenchymal stem cells. Stem Cells.

[66] Polchert D, Sobinsky J, Douglas G (2008). IFN-γ activation of mesenchymal stem cells for treatment and prevention of graft versus host disease. Eur J Immunol.

[67] Kim DS, Jang IK, Lee MW (2018). Enhanced immunosuppressive properties of human mesenchymal stem cells primed by interferon-γ. EBioMedicine.

[68] Abreu SC, Antunes MA, Xisto DG (2017). Bone marrow, adipose, and lung tissue-derived murine mesenchymal stromal cells release different mediators and differentially affect airway and lung parenchyma in experimental asthma. Stem Cells Transl Med.

[69] Guiducci S, Manetti M, Romano E, et al. Bone marrow-derived mesenchymal stem cells from early diffuse systemic sclerosis exhibit a paracrine machinery and stimulate angiogenesis in vitro. Ann Rheum Dis. 2011;70:2011–21. 10.1136/ard.2011.150607 21821866

[70] Maria ATJ, Rozier P, Fonteneau G (2018). iNOS activity is required for the therapeutic effect of mesenchymal stem cells in experimental systemic sclerosis. Front Immunol.

[71] Sandner P, Becker-Pelster E-M, Stasch JP (2018). Discovery and development of sGC stimulators for the treatment of pulmonary hypertension and rare diseases. Nitric Oxide Biol Chem.

[72] Chen X, Gan Y, Li W (2014). The interaction between mesenchymal stem cells and steroids during inflammation. Cell Death Dis.

[73] Ferrini MG, Vernet D, Magee TR (2002). Antifibrotic role of inducible nitric oxide synthase. Nitric Oxide Biol Chem.

[74] Filippin LI, Cuevas MJ, Lima E (2011). Nitric oxide regulates the repair of injured skeletal muscle. Nitric Oxide Biol Chem.

[75] Jiang M, Yu Y, Luo J, et al. Bone marrow-derived mesenchymal stem cells expressing thioredoxin 1 attenuate bleomycin-induced skin fibrosis and oxidative stress in scleroderma. J Investig Dermatol. 2017;137:1223–33. 10.1016/j.jid.2017.01.011 28132855

[76] Wu X, Li Y, Jin J (2020). Therapeutic effect of hypoxia-pretreated adipose tissue-derived stromal cells on mice with scleroderma. J Tongji Univ (Med Sci).

[77] Servettaz A, Goulvestre C, Kavian N, et al. Selective oxidation of DNA topoisomerase 1 induces systemic sclerosis in the mouse. J Immunol. 2009;182:5855–64. 10.4049/jimmunol.0803705 19380834

[78] Scuderi N, Ceccarelli S, Onesti MG, et al. Human adipose-derived stromal cells for cell-based therapies in the treatment of systemic sclerosis. Cell Transplant. 2013;22:779–95. 10.3727/096368912X639017 22526170

[79] Cras A, Farge D, Carmoi T, et al. Update on mesenchymal stem cell-based therapy in lupus and scleroderma. Arthritis Res Ther. 2015;17:301. 10.1186/s13075-015-0819-7 PMC4631077 26525582

[80] Rozier P, Maumus M, Maria ATJ, et al. Mesenchymal stromal cells-derived extracellular vesicles alleviate systemic sclerosis via miR-29a-3p. J Autoimmun. 2021;121:102660. 10.1016/j.jaut.2021.102660 34020253

[81] Kawashita Y, Jinnin M, Makino T (2011). Circulating miR-29a levels in patients with scleroderma spectrum disorder. J Dermatol Sci.

[82] Honda N, Jinnin M, Kajihara I, et al. TGF-β-mediated downregulation of microRNA-196a contributes to the constitutive upregulated type I collagen expression in scleroderma dermal fibroblasts. J Immunol. 2012;188:3323–31. 10.4049/jimmunol.1100876 22379029

[83] Baral H, Uchiyama A, Yokoyama Y, et al. Antifibrotic effects and mechanisms of mesenchymal stem cell-derived exosomes in a systemic sclerosis mouse model: possible contribution of miR-196b-5p. J Dermatol Sci. 2021;104:39–47. 10.1016/j.jdermsci.2021.08.006 34479773

[84] Benedetto PD, Liakouli V, Ruscitti P, et al. Blocking CD248 molecules in perivascular stromal cells of patients with systemic sclerosis strongly inhibits their differentiation toward myofibroblasts and proliferation: a new potential target for antifibrotic therapy. Arthritis Res Ther. 2018;20:223. 10.1186/s13075-018-1719-4 PMC6235209 30285896

[85] Harmanci D, Erkan EP, Kocak A, et al. Role of the microRNA-29 family in fibrotic skin diseases (review). Biomed Rep. 2017;6:599–604. 10.3892/br.2017.900 PMC5449962 28584629

[86] Ciechomska M, O’Reilly S, Suwara M (2014). MiR-29a reduces TIMP-1 production by dermal fibroblasts via targeting TGF-β activated kinase 1 binding protein 1, implications for systemic sclerosis. PLoS ONE.

[87] Cui J, Jin L, Ding M (2022). Efficacy and safety of mesenchymal stem cells in the treatment of systemic sclerosis: a systematic review and meta-analysis. Stem Cell Res Ther.

[88] Rockel JS, Rabani R, Viswanathan S. Anti-fibrotic mechanisms of exogenously-expanded mesenchymal stromal cells for fibrotic diseases. Semin Cell Dev Biol. 2019;101:87–103. 10.1016/j.semcdb.2019.10.014 31757583

[89] Granel B, Daumas A, Jouve E, et al. Safety, tolerability and potential efficacy of injection of autologous adipose-derived stromal vascular fraction in the fingers of patients with systemic sclerosis: an open-label phase I trial. Ann Rheum Dis. 2015;74:2175–82. 10.1136/annrheumdis-2014-205681 PMC4680117 25114060

[90] Keyszer GM, Christopeit M, Fick S, et al. Treatment of severe progressive systemic sclerosis with transplantation of mesenchymal stromal cells from allogeneic related donors: report of five cases. Arthritis Rheum. 2011;63:2540–2. 10.1002/art.30431 21547891

[91] Christopeit M, Schendel M, Föll J, et al. Marked improvement of severe progressive systemic sclerosis after transplantation of mesenchymal stem cells from an allogeneic haploidentical-related donor mediated by ligation of CD137L. Leukemia. 2008;22:1062–4. 10.1038/sj.leu.2404996 17972956

[92] Guiducci S, Porta F, Saccardi R, et al. Autologous mesenchymal stem cells foster revascularization of ischemic limbs in systemic sclerosis: a case report. Ann Intern Med. 2010;153:650–4. 10.7326/0003-4819-153-10-201011160-00007 21079220

[93] Loisel S, Lansiaux P, Rossille D (2023). Regulatory B cells contribute to the clinical response after bone marrow-derived mesenchymal stromal cell infusion in patients with systemic sclerosis. Stem Cells Transl Med.

[94] Farge D, Loisel S, Resche-Rigon M (2022). Safety and preliminary efficacy of allogeneic bone marrow-derived multipotent mesenchymal stromal cells for systemic sclerosis: a single-centre, open-label, dose-escalation, proof-of-concept, phase 1/2 study. Lancet Rheumatol.

[95] Boberg E, Bahr L von, Afram G, et al. Treatment of chronic GvHD with mesenchymal stromal cells induces durable responses: a phase II study. Stem Cells Transl Med. 2020;9:1190–202. 10.1002/sctm.20-0099 PMC7519760 32573983

[96] Li C, Wei S, Xu Q (2022). Application of ADSCs and their exosomes in scar prevention. Stem Cell Rev Rep.

[97] Wu Y, Huang S, Huang S, et al. Bone marrow‐derived mesenchymal stem cell attenuates skin fibrosis development in mice. Int Wound J. 2014;11:701–10. 10.1111/iwj.12034 PMC7950597 23409729

[98] Serratrice N, Bruzzese L, Magalon J, et al. New fat-derived products for treating skin-induced lesions of scleroderma in nude mice. Stem Cell Res Ther. 2014;5:138. 10.1186/scrt528 PMC4446000 25519759

[99] Chen B, Wang X, Long X (2018). Supportive use of adipose-derived stem cells in cell-assisted lipotransfer for localized scleroderma. Plast Reconstr Surg.

[100] Wernicke CM, Grunewald TG, Hendrik J (2011). Mesenchymal stromal cells for treatment of steroid-refractory GvHD: a review of the literature and two pediatric cases. Int Arch Med.

[101] Allison M (2009). Genzyme backs Osiris, despite Prochymal flop. Nat Biotechnol.

[102] Fujita Y, Kadota T, Araya J (2018). Clinical application of mesenchymal stem cell-derived extracellular vesicle-based therapeutics for inflammatory lung diseases. J Clin Med.

[103] Jayaram P, Ikpeama U, Rothenberg JB (2019). Bone marrow-derived and adipose-derived mesenchymal stem cell therapy in primary knee osteoarthritis: a narrative review. PM R.

[104] Kern S, Eichler H, Eichler H, et al. Comparative analysis of mesenchymal stem cells from bone marrow, umbilical cord blood, or adipose tissue. Stem Cells. 2006;24:1294–301. 10.1634/stemcells.2005-0342 16410387

[105] Liao H-T, Chen C-T (2014). Osteogenic potential: comparison between bone marrow and adipose-derived mesenchymal stem cells. World J Stem Cells.

[106] Ikegame Y, Yamashita K, Hayashi S-I (2011). Comparison of mesenchymal stem cells from adipose tissue and bone marrow for ischemic stroke therapy. Cytotherapy.

[107] Li C, Wu X, Tong J (2015). Comparative analysis of human mesenchymal stem cells from bone marrow and adipose tissue under xeno-free conditions for cell therapy. Stem Cell Res Ther.

[108] Roszek K, Wujak M (2018). How to influence the mesenchymal stem cells fate? Emerging role of ectoenzymes metabolizing nucleotides. J Cell Physiol.

[109] Peng L, Jia Z, Yin X (2008). Comparative analysis of mesenchymal stem cells from bone marrow, cartilage, and adipose tissue. Stem Cells Dev.

[110] Griffin M, Ryan CM, Pathan O, et al. Characteristics of human adipose derived stem cells in scleroderma in comparison to sex and age matched normal controls: implications for regenerative medicine. Stem Cell Res Ther. 2017;8:23. 10.1186/s13287-016-0444-7 PMC5297142 28173869

[111] Benedetto PD, Panzera N, Cipriani P, et al. Mesenchymal stem cells of Systemic Sclerosis patients, derived from different sources, show a profibrotic microRNA profiling. Sci Rep. 2019;9:7144. 10.1038/s41598-019-43638-0 PMC6509164 31073190

[112] Peltzer J, Peltzer J, Peltzer J, et al. Mesenchymal stromal cells based therapy in systemic sclerosis: rational and challenges. Front Immunol. 2018;9:2013. 10.3389/fimmu.2018.02013 PMC6146027 30271402

[113] Andrews S, Maughon T, Marklein R (2021). Priming of MSCs with inflammation-relevant signals affects extracellular vesicle biogenesis, surface markers, and modulation of T cell subsets. J Immunol Regen Med.

[114] Wang L, Gu Z, Zhao X (2016). Extracellular vesicles released from human umbilical cord-derived mesenchymal stromal cells prevent life-threatening acute graft-versus-host disease in a mouse model of allogeneic hematopoietic stem cell transplantation. Stem Cells Dev.

[115] Murphy MB, Moncivais K, Caplan AI (2013). Mesenchymal stem cells: environmentally responsive therapeutics for regenerative medicine. Exp Mol Med.

[116] Aggarwal S, Pittenger MF (2005). Human mesenchymal stem cells modulate allogeneic immune cell responses. Blood.

[117] Bartholomew A, Sturgeon C, Siatskas M (2002). Mesenchymal stem cells suppress lymphocyte proliferation in vitro and prolong skin graft survival in vivo. Exp Hematol.

[118] Von Lüttichau I, Notohamiprodjo M, Wechselberger A (2005). Human adult CD34- progenitor cells functionally express the chemokine receptors CCR1, CCR4, CCR7, CXCR5, and CCR10 but not CXCR4. Stem Cells Dev.

[119] Cipriani P, Di Benedetto P, Dietrich H (2016). Searching for a good model for systemic sclerosis: the molecular profile and vascular changes occurring in UCD-200 chickens strongly resemble the early phase of human systemic sclerosis. Arch Med Sci AMS.

[120] Jiménez SA, Castro SV, Piera-Velázquez S (2010). Role of growth factors in the pathogenesis of tissue fibrosis in systemic sclerosis. Curr Rheumatol Rev.

[121] Li X, Li Z, Liu H (2022). Rituximab combined with mesenchymal stem cells for the treatment of sclerodermatous chronic graft-versus-host disease: a case report. Hainan Med J.

[122] Chen C, Wang D, Moshaverinia A, et al. Mesenchymal stem cell transplantation in tight-skin mice identifies miR-151-5p as a therapeutic target for systemic sclerosis. Cell Res. 2017;27:559–77. 10.1038/cr.2017.11 PMC5385608 28106077

[123] Chia JJ, Zhu T, Chyou S, et al. Dendritic cells maintain dermal adipose–derived stromal cells in skin fibrosis. J Clin Investig. 2016;126:4331–45. 10.1172/JCI85740 PMC5096920 27721238

[124] Zu Y, Zhou J, Fu Y (2021). Feasibility of reduced-dose posttransplant cyclophosphamide and cotransplantation of peripheral blood stem cells and umbilical cord-derived mesenchymal stem cells for SAA. Sci Rep.

[125] Xu J, Li L, Xiong J (2015). Cyclophosphamide combined with bone marrow mesenchymal stromal cells protects against bleomycin-induced lung fibrosis in mice. Ann Clin Lab Sci.

[126] Hajkova M, Jaburek F, Porubska B (2019). Cyclosporine A promotes the therapeutic effect of mesenchymal stem cells on transplantation reaction. Clin Sci.

[127] Hajkova M, Hermankova B, Javorkova E (2017). Mesenchymal stem cells attenuate the adverse effects of immunosuppressive drugs on distinct T cell subopulations. Stem Cell Rev Rep.

[128] Hajkova M, Javorkova E, Zajicova A (2017). A local application of mesenchymal stem cells and cyclosporine A attenuates immune response by a switch in macrophage phenotype: MSCs induce immunosuppressive macrophages in vivo. J Tissue Eng Regen Med.

[129] Shi D, Liao L, Zhang B (2011). Human adipose tissue-derived mesenchymal stem cells facilitate the immunosuppressive effect of cyclosporin A on T lymphocytes through Jagged-1-mediated inhibition of NF-κB signaling. Exp Hematol.

[130] Kato T, Khanh VC, Sato K (2018). Elevated expression of Dkk-1 by glucocorticoid treatment impairs bone regenerative capacity of adipose tissue-derived mesenchymal stem cells. Stem Cells Dev.

[131] Zhou D-A, Zheng H-X, Wang C-W (2014). Influence of glucocorticoids on the osteogenic differentiation of rat bone marrow-derived mesenchymal stem cells. BMC Musculoskelet Disord.

[132] Kemp K, Morse R, Sanders K (2011). Alkylating chemotherapeutic agents cyclophosphamide and melphalan cause functional injury to human bone marrow-derived mesenchymal stem cells. Ann Hematol.

[133] Tsuji W, Schnider JT, McLaughlin MM (2015). Effects of immunosuppressive drugs on viability and susceptibility of adipose- and bone marrow-derived mesenchymal stem cells. Front Immunol.

[134] Schneider N, Gonçalves FDC, Pinto FO (2015). Dexamethasone and azathioprine promote cytoskeletal changes and affect mesenchymal stem cell migratory behavior. PLoS ONE.

[135] Javorkova E, Vackova J, Hajkova M (2018). The effect of clinically relevant doses of immunosuppressive drugs on human mesenchymal stem cells. Biomed Pharmacother.

[136] Lee HK, Kim KH, Kim HS (2018). Effect of a combination of prednisone or mycophenolate mofetil and mesenchymal stem cells on lupus symptoms in MRL. *Fas*^lpr^ mice. Stem Cells Int.

[137] Li Z, Chen S, Ma K (2018). CsA attenuates compression-induced nucleus pulposus mesenchymal stem cells apoptosis via alleviating mitochondrial dysfunction and oxidative stress. Life Sci.

[138] Lee NK, Myeong SH, Hwang JW (2022). Combination of dexamethasone and tofacitinib reduces xenogeneic MSC-induced immune responses in a mouse model of Alzheimer’s disease. Biomedicines.

[139] Hwang JW, Myeong SH, Lee N-H (2021). Immunosuppressant drugs mitigate immune responses generated by human mesenchymal stem cells transplanted into the mouse parenchyma. Cell Transplant.

[140] Wang L, Fan J, Lin Y-S (2015). Glucocorticoids induce autophagy in rat bone marrow mesenchymal stem cells. Mol Med Rep.

